# Physiological responses and drought resistance evaluation of forage oat (*Avena sativa* L.) seedling cultivar under drought stress

**DOI:** 10.1080/15592324.2025.2574391

**Published:** 2025-11-04

**Authors:** Ting Song, Jiahui Hang, Xiaotong Shi, Xingcai Liu, Dongmei Ma

**Affiliations:** a College of Eco-Environment, NingXia University, Yinchuan, People's Republic of China; b College of Life Sciences, NingXia University, Yinchuan, People's Republic of China

**Keywords:** Forage oat, drought resistance, principal component analysis, cluster analysis, affiliation function approach

## Abstract

Oat (*Avena sativa* L.) is an important forage crop widely used in animal husbandry. However, the greenhouse effect, which leads to increasing global temperatures, extreme water scarcity, and more frequent drought events, also creates abiotic stress that inhibits oat growth. Drought stress strongly affects the yield and quality of forage oats, hindering the selection, promotion, and utilization of drought-resistant cultivars. This study investigated alterations in the growth and physiological traits of diverse oat cultivars under drought stress for varying durations. We comprehensively assessed the drought resistance capabilities of each variety. Forty oat cultivars were subjected to drought stress starting from plant growth up to the two-leaf stage a pot-based water withholding method. The stress durations were 0 d, 7 d, and 14 d. Compared with those of the control, the key physiological parameters of the test cultivar decreased with increasing drought stress duration. These factors increased the maximum photochemical quantum yield (Fv/Fm), PS II quantum efficiency (Fv/Fo), soil plant analysis development (SPAD) value, net photosynthetic rate (Pn), transpiration rate (Tr), and stomatal conductance (Gs). Conversely, the malondialdehyde (MDA) and proline (Pro) contents increased. Antioxidant enzyme activity initially increased but subsequently decreased. Changes in osmoregulatory substance content and the modulation of antioxidant enzyme activity are key components of drought resistance mechanisms. Therefore, Fv/Fo, Pro, Tr, Gs, and Pn have emerged as reliable parameters for assessing drought resistance in forage oat seedlings. When assessing seedling drought resistance using biochemical parameters such as photosynthesis, a comprehensive analysis combining multiple indicators and methods is essential. This study provides a theoretical basis for screening drought-resistant oat cultivars and for high-yield cultivation practices.

## 1 Introduction

Oats (*Avena sativa* L.) are annual cool-season herbaceous plants of the genus oat in the family Gramineae and are widely cultivated and used worldwide both as grains and as fodder.^1^^,^^2^ Oats are widely adaptable, with excellent characteristics such as cold resistance, drought resistance, and salinity resistance. Oats grow mainly in agricultural and pastoral areas in northern China and serve as the main grain crop and feed crop in the northern region.^3^ Oats are subjected to many abiotic stresses during growth,^4^ of which drought stress is one of the major factors limiting their growth.^5^^,^^6^ Arid environments severely constrain plant seed germination,^7^^,^^8^ plant growth and development,^9^^,^^10^ and crop yield,^11^^,^^12^ the main manifestations of which are water utilization and partitioning in different organs, inhibition of leaf and root growth, impairment of cell membrane permeability, reduction in osmotic potential, reduction in photosynthesis, and excessive production of reactive oxygen species (ROS). These factors accelerate the abnormal yellowing and shedding of leaves when plants are subjected to drought stress.^13^ However, as sessile organisms, plants cannot replace their growing environment to completely escape adversity stress. For this reason, plants employ a series of adaptive strategies, including growth patterns, physiological mechanisms and molecular level changes,^14^ which enable them to adapt to and mitigate the effects of adversity through adaptive structure formation, physiological process regulation, and signaling and gene regulation, thereby safeguarding their ability to survive and reproduce.

The seedling stage is the most sensitive period in terms of plant growth and development. Germination is a critical period in the growth and development of plants. When plants are subjected to drought stress during the germination period, the germination rate is affected,^15^^,^^16^ reducing seedling emergence and lowering the production yield of the plant at the seedling stage. Drought stress inhibits plant growth and negatively affects plant yield and physiological and biochemical characteristics,^17^ with plants showing different resistance levels.

Currently, the identification of oat drought resistance lies in the germination period. Researchers have reported that 15% polyethylene glycol (PEG) inhibits the germination of oat seeds. The contents of malondialdehyde (MDA) and soluble sugar (SS) increase with increasing duration of drought stress, and the activities of antioxidant enzymes also increase.^18^ Compared with the germination stage, the seedling stage of oat also enables visualization of the phenotype and yield of oat under drought stress and accurate determination of the efficiency of photosynthesis, antioxidant enzyme activity and osmoregulatory substances. Therefore, analyzing the different responses of different oat cultivars under different durations of drought stress during the seedling stage is important for identifying drought-tolerant cultivars.

To avoid the limitations and contingencies of single index measurements, the current comprehensive evaluation of plant stress tolerance is commonly based on a combination of principal component analysis (PCA) and affiliation function analysis and cluster analysis followed by a comprehensive evaluation.^19-21^ Comprehensive evaluation methods are widely used to identify and evaluate stress tolerance in many plants.^22-24^ There are few reports on the identification and evaluation of drought resistance in oats under different durations of drought stress.

Forage oats are highly important in the animal husbandry, agriculture, ecology, and socioeconomic sectors. However, due to water scarcity caused by global warming and the unique geographical environments in which they are cultivated, the growth of forage oats is highly susceptible to drought stress, which affects yield and crop quality. To address this issue, in this study, 40 cultivars of forage oat were selected and subjected to the potting water control method to study the response of different drought-resistant types to different durations of drought. Their level of drought resistance was identified and comprehensively evaluated using correlation analysis, principal component analysis, cluster analysis, and the affiliation function method. The purpose of this study was to select high-quality drought-resistant oat cultivars and provide a basis for the identification of drought resistance indices and comprehensive evaluation methods for oat.

## 2 Materials and methods

### 2.1 Plant materials and cultivation conditions

Plant cultivars were obtained from the seed bank of the College of Eco-Environment, Ningxia University, Yinchuan, China. Table 1 lists the 40 forage oat accessions used in the drought stress experiment.

**Table 1.** The origin of 40 accessions of oats.

Number	Variety name	Country of origin	Number	Variety name	Country of origin	Number	Variety name	Country of origin	Number	Variety name	Country of origin
S1	Qing yongjiu741	China	S11	OA123-81	China	S21	Mulga Kansas	China	S31	Fulghum	China
S2	Mc0912	China	S12	SANXING	China	S22	Baiyan No.7	China	S32	Molasses	American
S3	Jime Oat	China	S13	O.T.432	China	S23	Dictator	American	S33	Ot620	China
S4	Ever leaf26	American	S14	Tianyan No.1	China	S24	Qing yongjiu402	China	S34	Lanark	China
S5	PG3	China	S15	Erban	China	S25	Qing yongjiu311	China	S35	Baler	Canada
S6	Souris	Canada	S16	Patterson	China	S26	PG2	China	S36	M151-1	China
S7	Pendek-Pc-48	China	S17	Jasiri	China	S27	PG8	China	S37	FF64-74	China
S8	OA291-10	China	S18	OA395-5	China	S28	Avoine 236	China	S38	Mabel	China
S9	Minrus	China	S19	PC56	China	S29	Obec 033	China	S39	Acton	China
S10	Aa68-75	China	S20	Montcalm	China	S30	Pearle (Perle)	China	S40	Avoine 125	China

The experiment was conducted in 2023 at the Artificial Climate Laboratory, College of Ecology and Environment, Ningxia University. For the pot water control method, loess and nutrient soil were mixed at a ratio of 1:1 and filled into perforated plastic pots with an outer diameter of 24 cm, an inner diameter of 22 cm, and a height of 19 cm for seedling cultivation. Each pot was filled with 70% mixed soil. Forty plump seeds of each oat variety were selected for sowing. During the seedling emergence period, water was regularly supplied, and the indoor temperature was controlled to fluctuate between 24 °C and 26 °C.

### 2.2 Methods

When the plants grew to the two-leaf-one-heart stage, to avoid insufficient test materials in the later stage due to long-term drought, 30 seedlings with consistent growth vigor were retained in each pot. Before the drought treatment, the experimental materials were fully irrigated for three consecutive days until the water seeped out of the trays at the bottom of the flowerpots to ensure a uniform soil moisture content in the experimental area. On the day after the third irrigation, except for the control group, which was watered normally, the treatment groups were subjected to natural drought treatment. The soil water saturation content was determined by measuring the weight of the pots; these values were used to control the soil moisture capacity according to the experimental requirements. Three drought gradients were set: the soil of the control group contained 70−85% of the maximum water holding capacity; mild drought was typically attained after approximately 7 d of water withholding (with a relative soil water content of 50%–60% of the field maximum water-holding capacity), while severe drought required approximately 14 d (with a relative soil water content of 30%–40% of the field maximum water-holding capacity). By reducing the irrigation amount, the soil water content of the mixed soil in the flowerpots reached different drought gradients, and the natural drought duration was determined. Each treatment had three replicates. After 0 d, 7 d, and 14 d of drought treatment, at 10:00 am on the same day of drought, oat seedlings under drought stress were randomly selected for measurement. The chlorophyll content was measured on the second leaf counted from the top of the stem, and the physiological indexes were measured on the two to four leaves counted from the top of the stem (after the leaves were removed, they were immediately stored in a −80 °C refrigerator). The experiment had three replicates.

In this study, a total of 10,800 oat plants were cultivated. For the 40 oat varieties, 90 plants were subjected to the control treatment (well-watered), 90 plants were subjected to mild drought stress, and 90 plants were subjected to severe drought stress. This design provided *n* = 5 biological replicates per variety per treatment condition.

#### 2.2.1 Determination of growth and photosynthetic parameters

(1) Plant height (PH, cm): The length from the base of the stem to the top of the plant was measured using a ruler.
(2) Leaf length (LL, cm): The length from the tip of the leaf blade to the leaf sheath of the penultimate leaf of the plant was measured using a ruler.
(3) Leaf width (LW, cm): The widest part of the penultimate leaf blade was measured using a ruler.
(4) Leaf area (LA, cm^2^): Leaf length (cm) × Leaf width (cm) × 0.73, where 0.73 is the correction factor of the leaf area of forage oat.
(5) Shoot biomass (SB, g/plant), Root biomass (RB, g/plant): Single plants were sampled after measuring indicators such as plant height. The root systems were rinsed with distilled water and then dried. The shoots and roots were separated and placed in an oven at 105 ± 2 °C for 30 minutes. They were then dried at 80 ± 2 °C until a constant weight was achieved. The shoots and roots were weighed separately. The shoot biomass and root biomass were calculated and expressed as dry weight per plant.
(6) Root-to-shoot ratio (RSR): The root dry weight of the treatment/stem and leaf dry weight of the treatment × 100%.
(7) Relative water content (RWC): Leaves were picked from the same parts of the plants, and this operation was repeated five times. The leaves were weighed to record their fresh weight (FW). The leaves were subsequently soaked in distilled water at 4 °C in the dark for 24 hours, after which the turgid weight (TW) was measured. The leaves were subsequently placed in an aluminum box, first baked in an oven at 105 °C for 30 minutes, and then baked in an oven at 65 °C for 24 hours until a constant weight was reached. After that, the leaves were weighed again to record their dry weight (DW).

Relative water content = [(FW − DW)/(TW − DW)] × 100%

(8) Determination of photosynthetic parameters of plant leaves: Using the LI-6400 portable photosynthesis system, the following gas exchange parameters were measured during clear, rain-free mornings (9:00–12:00) at each developmental stage:
  1. Net photosynthetic rate (Pn, μmol·m^−2^·s^−1^);
  2. Transpiration rate (Tr, mmol·m^−2^·s^−1^);
  3. Stomatal conductance (Gs, mmol·m^−2^·s^−1^).
(9) Chlorophyll content (chlorophyll, Chl): A portable chlorophyll meter (SPAD-502) was used for measurement. This instrument indirectly calculates the chlorophyll content, expressed as the soil-plant analysis development (SPAD) value, by measuring the absorption and transmission of light at specific wavelengths (650 nm and 940 nm) by the leaves.
(10) Chlorophyll fluorescence parameters: Chlorophyll fluorescence was determined using a JC-YG 1162 chlorophyll fluorometer instrument. Before the measurement, the leaves were dark-adapted with dark reaction clips for 20 min, and the initial fluorescence (Fo) and maximum fluorescence (Fm) were measured. The maximum photochemical quantum yield (Fv/Fm) and PS II quantum efficiency (Fv/Fo) were derived from the measurements.

#### 2.2.2 Comprehensive evaluation of drought resistance

First, a correlation analysis was performed between the six growth indices of the forage oat at the seedling stage under drought stress. The six indicators included (1) plant height, (2) leaf area, (3) shoot biomass, (4) root biomass, (5) the root-to-shoot ratio, and (6) the relative water content. The six indicators at the seedling stage of the 40 forage oat materials were subsequently downgraded by principal component analysis, and the comprehensive drought resistance indicators were screened out. Finally, the drought resistance was comprehensively evaluated by the affiliation function and systematic clustering methods.^25^

Value of the affiliation function of the composite indicator for each material:

(1) $$U\;(X_{j})\;=\;(X_{j}—X_{\min})/(X_{\max}—X_{\min}),\;j\;=\;1,\;2,\;3,\;\ldots ,\;n$$

where X_j_ denotes the jth composite indicator, X_min_ denotes the minimum value of the jth composite indicator and X_max_ denotes the maximum value of the jth composite indicator.

Weighting of composite indicators for each material:

(2) $$W_{j}\;=\;P_{j}/\sum_{j}\;=\;1n_{\mathrm{Pj}},\;j\;=\;1,\;2,\;3\;\ldots ,\;n$$

where W_j_ denotes the importance of the jth composite indicator among all composite indicators and P_j_ is the contribution of the jth composite indicator for each genotype.

Comprehensive evaluation of drought resistance of each material:

(3) $$D\;=\;\sum_{j}\;=\;1n_{\left[U\;(\mathrm{Xj})\;\times \;\mathrm{Wj}\right]},\;j=\;1,\;2,\;3,\;\ldots ,\;n$$

The D-value is the combined evaluation value of drought resistance for each genotype under drought stress conditions obtained from the composite index evaluation.

#### 2.2.3 Determination of biochemical parameters

Morphological growth indices such as plant height, leaf area, shoot biomass, root biomass, root-to-shoot ratio, and relative water content were measured on an initial panel of 40 forage oat lines under drought stress. Based on these combined measurements, a comprehensive drought tolerance evaluation using D-value calculation and cluster analysis was conducted. This analysis identified five distinct lines representing a spectrum of drought tolerance levels of highly drought-resistant plants, moderately resistant plants, low-resistance plants, low-susceptibility plants, and highly susceptible plants. These five selected lines were then utilized for subsequent biochemical analyses, including biochemical assays and photosynthesis and chlorophyll fluorescence parameters.

The activity of superoxide dismutase (SOD, U/g FW) was determined by the nitro-blue tetrazolium (NBT) photoreduction method. The principle is that SOD inhibits the photoreduction of NBT, and SOD activity is calculated by detecting the change in absorbance of the reaction system at a wavelength of 560 nm.^26^​​​​​ The activity of peroxidase (POD, U/g FW) was measured using the guaiacol method. POD catalyzes the reaction between hydrogen peroxide and guaiacol to produce a reddish-brown substance. The POD activity was determined by measuring the change in absorbance at 470 nm.^27^ The activity of catalase (CAT, U/g FW) was calculated by measuring the rate of decrease in the absorbance of hydrogen peroxide at a wavelength of 240 nm.^28^ The proline (Pro) content was determined by the acidic ninhydrin method. Under acidic conditions, proline reacts with ninhydrin to form a stable red compound, and the content is measured colorimetrically using a spectrophotometer at a wavelength of 520 nm.^29^ The malondialdehyde (MDA) content is mainly determined by the thiobarbituric acid (TBA) colorimetric method. Under acidic and high-temperature conditions, MDA can react with TBA to form a reddish-brown trimethine (3,5,5-trimethyloxazole-2,4-dione), which has a maximum absorption peak at a wavelength of 532 nm and a relatively small absorption peak at 600 nm. By measuring the absorbance at 532 nm and 600 nm and using a specific formula to calculate the MDA content, the interference of other substances can be eliminated, thus accurately reflecting the MDA level in the sample.^30^

### 2.3 Data analysis

All experimental data were collated using Excel 2019. The data were analyzed by analysis of variance, correlation analysis, PCA, and cluster analysis using SPSS 26.0 and Origin 2022. All the statistical analyses were conducted using spss 26.0 software. Data normality and homogeneity of variance were verified using Shapiro-Wilk test and Levene's test. Differences between varieties under the same treatment were assessed using one-way analysis of variance (ANOVA). When ANOVA revealed significant differences (*P* < 0.05), post hoc multiple comparisons were performed using Duncan's multiple range test.

## 3 Results

### 3.1 Analysis of variance of oat under drought stress

The results of the ANOVA for various traits of 40 forage oat accessions (Table 2) revealed that, except for the relative water content (RWC) under 7 d of drought stress, which reached a significant level among accessions, the differences in all other traits reached a highly significant level among accessions. This indicates that there is extensive variation in the traits of different forage oat accessions.

**Table 2.** Variance analysis of different characteristics among provenances of oat.

Traits	Drought treatment for 0 d			Drought treatment for 7 d			Drought treatment for 14 d		
	Degrees of freedom	*F*-value	*P*-value	Degrees of freedom	*F*-value	*P*-value	Degrees of freedom	*F*-value	*P*-value
PH	39	30.729	0.000	39	26.181	0.000	39	32.135	0.000
LA	39	26.956	0.000	39	27.037	0.000	39	27.401	0.000
SB	39	20.917	0.000	39	18.154	0.000	39	21.465	0.000
RB	39	10.563	0.000	39	10.783	0.000	39	10.578	0.000
RSR	39	11.304	0.000	39	9.430	0.000	39	14.047	0.000
RWC	39	8.109	0.000	39	1.435	0.051	39	8.836	0.000

### 3.2 Growth morphological responses of oat to drought stress

**Figure 1.**
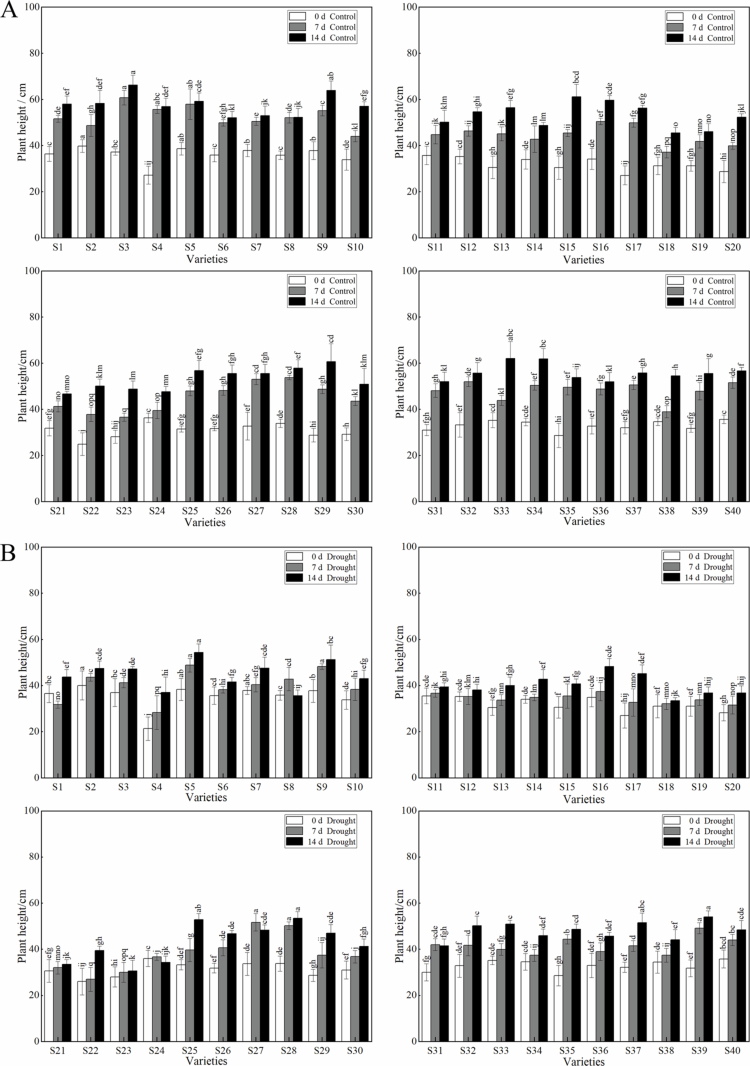
Effect of drought stress on plant height of different varieties of forage oat. Note: **(A)** represents the plant height of the control. **(B)** represents the height of the plants in the natural drought treatment groups. White color represents 0 d of drought stress. Gray color represents 7 d of drought stress. Black color represents 14 d of drought stress. Bars with different lowercase letters are significantly different at *P* < 0.05, and values represent the mean dry weight per plant ± standard error (Duncan's multiple range test).

Plant height (PH) can reflect whether plant growth and development are healthy and complete. It is also an important indicator of production capacity as a way to visualize a plant's pasture yield. The PH of all the forage oat cultivars under no drought stress treatment (control) increased significantly during the incubation period (Figure 1), with the highest value for variety S30 and the lowest value for variety S21, which were significantly different (*P* < 0.05). Under drought-stress conditions, S3 had the greatest PH, and S18 had the smallest PH, which were significantly different (*P* < 0.05). Under drought stress, there were only eight cultivars with plant heights above 50 cm: S9, S25, S28, S32, S33, S37, and S39. Five cultivars, S5, S25, S28, S37, and S39, were not significantly different in height from the control, but the remaining 35 cultivars had significantly different plant heights than the control. Under drought conditions, the plant heights all decreased to different degrees. The most affected by drought stress was S37, with a decrease of 37.24%, and the least affected by drought stress was S39; with a decrease of 2.8%, this variety showed extreme drought resistance.

The leaf area (LA) reflects the magnitude of a plant's photosynthetic rate; the larger the effective LA is, the greater the plant's ability to photosynthesize. As shown in Table 3, the differences in the leaf area of the different forage oat cultivars between each treatment were significant (*P* < 0.05). As the duration of drought stress increased, the leaf area of the plants in the treatment group, although it tended to increase, decreased compared with that of the control group, which affected the photosynthesis of the plants at a later stage. Under drought stress, variety S27 presented the greatest leaf area, and variety S22 presented the smallest LA. The difference in leaf area per leaf for S5, S28, S31, S35, S36, and S39 under drought stress was not significant compared with that of the control, indicating that drought did not have much effect on the leaf area of these cultivars; the growth of their leaves was not sensitive to drought stress.

**Table 3.** Effect of drought stress on leaf area per leaf of different cultivar of oat.

Cultivar	Control			Drought		
	0 d	7 d	14 d	0 d	7 d	14 d
S1	8.9 ± 0.39^abcde^	16.65 ± 0.18^cdefgh^	28.99 ± 0.58^cdefg^	9.58 ± 0.38^bcdefghi^	12.21 ± 0.26^hij^	15.34 ± 0.29^fgh^
S2	10.15 ± 0.33^a^	19.96 ± 0.57^cdefghi^	29.17 ± 0.93^cdefg^	14.01 ± 1.04^a^	18.58 ± 0.27^b^	22.41 ± 0.29^abcde^
S3	9.21 ± 0.17^abcd^	27.52 ± 2.82^b^	33.11 ± 0.72^a^	12.45 ± 1.01^abcd^	17.28 ± 0.43^bc^	20.99 ± 0.48^abcdefg^
S4	5.54 ± 0.47^ij^	28.88 ± 0.5^a^	32.48 ± 0.6^defgh^	4.65 ± 0.85^k^	11.27 ± 1.21^ij^	14.7 ± 1.29^gh^
S5	9.73 ± 0.33^ab^	28.49 ± 2.82^b^	29.59 ± 0.6^cdef^	13.15 ± 0.79^ab^	21.33 ± 0.47^a^	25.44 ± 0.5^abc^
S6	8.73 ± 0.36^abcde^	16.34 ± 0.2^cdefgh^	26.03 ± 0.45^jklm^	11.81 ± 0.63^abcdef^	14.5 ± 0.27^bcde^	20.42 ± 0.31^bcdefgh^
S7	9.45 ± 0.33^abc^	17.53 ± 0.35^defghijk^	26.5 ± 0.69^hijklm^	12.92 ± 0.29^abc^	17.64 ± 0.49^bc^	21.7 ± 0.53^abcdef^
S8	8.7 ± 0.22^abcde^	16.16 ± 0.34^cdefghijk^	26.14 ± 0.64^ijklm^	11.88 ± 0.35^abcde^	15.81 ± 0.83^b^	22.9 ± 0.89^abcd^
S9	9.42 ± 0.47^abc^	23.56 ± 0.34^c^	31.95 ± 0.68^ab^	12.84 ± 0.83^abc^	21.23 ± 0.26^a^	25.39 ± 0.3^abc^
S10	7.99 ± 0.56^abcdefgh^	18.68 ± 0.29^cdefghijk^	28.52 ± 0.32^defgh^	10.87 ± 0.67^abcdefgh^	16.5 ± 0.81^bcde^	20.39 ± 0.86^bcdefgh^
S11	8.66 ± 0.49^abcde^	15.33 ± 0.5^defghijk^	25.08 ± 0.86^lmno^	11.73 ± 0.57^abcdefg^	14.92 ± 0.28^cdefgh^	18.4 ± 0.31^defgh^
S12	8.49 ± 0.39^abcdef^	14.75 ± 0.27^cdefghijk^	27.32 ± 0.3^ghijk^	11.6 ± 0.34^abcdefg^	13.98 ± 0.54^efgh^	17.32 ± 0.58^defgh^
S13	6.77 ± 0.59^efghij^	14.39 ± 0.37^defghijk^	28.23 ± 0.52^efghij^	9.21 ± 0.56^cdefghi^	13.33 ± 0.4^fghi^	16.62 ± 0.43^defgh^
S14	8.02 ± 0.51^abcdefgh^	13.99 ± 0.7^efghijk^	24.36 ± 0.22^mnop^	11.02 ± 0.29^abcdefgh^	14.45 ± 0.26^defgh^	18.06 ± 0.32^defgh^
S15	6.74 ± 0.61^efghij^	14.16 ± 0.18^defghijk^	30.59 ± 0.89^bcd^	9.27 ± 0.77^cdefghi^	13.81 ± 0.86^efghi^	17.09 ± 0.96^defgh^
S16	8.1 ± 0.55^abcdefgh^	16.49 ± 0.2^cdefgh^	29.83 ± 0.34^cdef^	11.42 ± 0.67^abcdefg^	15.34 ± 0.72^cdefg^	18.87 ± 0.81^cdefgh^
S17	5.5 ± 0.5^ij^	16.16 ± 0.25^cdefgh^	28.12 ± 0.21^efghij^	7.51 ± 0.9^ij^	12.84 ± 0.99^ghij^	16.1 ± 1.08^efgh^
S18	7.02 ± 0.46^defghij^	11.69 ± 0.29^k^	22.77 ± 0.39^p^	9.51 ± 0.84^bcdefghij^	12.74 ± 0.37^ghij^	16.06 ± 0.4^efgh^
S19	7.02 ± 0.28^defghij^	13.72 ± 0.34^fghijk^	23.02 ± 0.56^op^	9.53 ± 0.71^bcdefghij^	13.97 ± 0.35^efgh^	17.58 ± 0.35^defgh^
S20	6.11 ± 0.59^ghij^	12.77 ± 0.2^hijk^	26.15 ± 0.25^ijklm^	8.1 ± 0.59^ghij^	12.59 ± 0.61^hij^	15.96 ± 0.65^efgh^
S21	7.23 ± 0.4^cdefghi^	13.55 ± 0.31^fghijk^	23.37 ± 0.18^nop^	9.31 ± 0.83^cdefghi^	13.14 ± 0.44^fghi^	16.71 ± 0.47^defgh^
S22	4.71 ± 0.6^j^	12.19 ± 0.37^ijk^	25.07 ± 0.49^lmno^	7.01 ± 0.97^j^	10.51 ± 0.85^j^	13.82 ± 0.91^h^
S23	5.89 ± 0.35^hij^	12.98 ± 0.25^ijk^	24.39 ± 0.58^mnop^	8.02 ± 0.72^hij^	12.33 ± 0.69^hij^	15.94 ± 0.72^efgh^
S24	8.88 ± 0.25^abcde^	15.01 ± 0.45^cdeghij^	23.82 ± 0.39^nop^	12.01 ± 0.59^abcde^	15.63 ± 0.26^cdef^	19.44 ± 0.28^bcdefgh^
S25	7.11 ± 0.16^cdefghi^	16.22 ± 0.26^cdefghi^	28.41 ± 0.73^efgh^	10.65 ± 0.37^abcdefghi^	16.99 ± 0.81^bcd^	20.86 ± 0.87^bcdefgh^
S26	7.19 ± 0.12^cdefghi^	17.91 ± 0.26^cdef^	27.77 ± 0.61^fghij^	9.93 ± 0.34^bcdefghi^	17.29 ± 0.5^bc^	21.09 ± 0.51^abcdefg^
S27	7.56 ± 0.73^bcdefghi^	25.08 ± 0.31^cd^	27.79 ± 0.66^fghij^	10.84 ± 0.83^abcdefgh^	23.24 ± 0.6^a^	27.61 ± 0.63^a^
S28	8.01 ± 0.22^abcdefgh^	17.21 ± 0.13^cdef^	28.95 ± 0.6^cdefg^	10.91 ± 0.54^abcdefgh^	15.2 ± 0.32^cdefg^	25.37 ± 0.35^abc^
S29	6.12 ± 0.36^ghij^	16.23 ± 0.3^cdefgh^	30.33 ± 1.29^bcde^	8.35 ± 0.43^fghij^	15.64 ± 0.92^cdef^	19.29 ± 0.99^bcdefgh^
S30	6.27 ± 0.33^fghij^	16.95 ± 0.28^cdefgh^	25.46 ± 1.13^klmn^	9.52 ± 0.66^bcdefghi^	15.37 ± 0.52^cdefg^	19.02 ± 0.59^cdefgh^
S31	9.94 ± 0.29^abc^	18.13 ± 0.33^cdefghi^	26.03 ± 0.67^jklm^	9.02 ± 0.62^defghi^	17.54 ± 0.42^bc^	21.24 ± 0.45^abcdefg^
S32	7.76 ± 0.65^bcdefghi^	17.72 ± 0.25^cde^	27.86 ± 0.76^fghij^	10.43 ± 0.82^abcdefghi^	17.53 ± 0.75^bc^	21.26 ± 0.82^abcdefg^
S33	8.49 ± 0.4^abcdef^	14.41 ± 0.33^defghijk^	31.03 ± 1.22^bc^	11.59 ± 0.31^abcdefg^	16.37 ± 0.5^bcde^	19.94 ± 0.55^bcdefgh^
S34	8.21 ± 0.2^abcdefgh^	17.12 ± 0.27^cdef^	30.95 ± 0.74^bc^	9.29 ± 0.6^abcdefg^	15.39 ± 0.38^cdefg^	18.95 ± 0.4^cdefgh^
S35	6.08 ± 0.64^ghij^	17.05 ± 0.44^cdef^	26.9 ± 0.63^ghijkl^	8.31 ± 0.74^fghij^	16.08 ± 0.31^bcd^	22.99 ± 0.34^abcd^
S36	7.57 ± 0.42^bcdefghi^	16.47 ± 0.27^cdefgh^	25.98 ± 0.66^jklm^	7.49 ± 0.87^ij^	16.41 ± 0.65^bcde^	20.1 ± 0.71^bcdefgh^
S37	7.32 ± 0.33^cdefghi^	16.74 ± 0.3^cdefg^	27.9 ± 0.37^fghij^	7.11 ± 0.37^bcdefghi^	15.66 ± 0.45^cdefg^	21.47 ± 0.52^abcdef^
S38	8.26 ± 0.2^abcdefg^	17.2 ± 0.33^cdef^	27.26 ± 0.48^ghijkl^	11.22 ± 0.8^abcdefgh^	15.32 ± 0.51^cdefg^	18.83 ± 0.56^cdefgh^
S39	7.23 ± 0.21^cdefghi^	22.82 ± 0.38^cd^	27.78 ± 1.06^fghij^	9.88 ± 0.6^bcdefghi^	21.6 ± 0.42^a^	25.76 ± 0.46^ab^
S40	8.65 ± 0.23^abcde^	19.93 ± 0.31^cdefg^	28.36 ± 0.25^efghi^	8.9 ± 0.64^fghij^	18.75 ± 0.4^b^	22.58 ± 0.44^abcde^

Note: Values with different superscript letters (a, b, c) within a row indicate significant differences (*P* < 0.05) across timepoints (Duncan's multiple range test).

**Figure 2.**
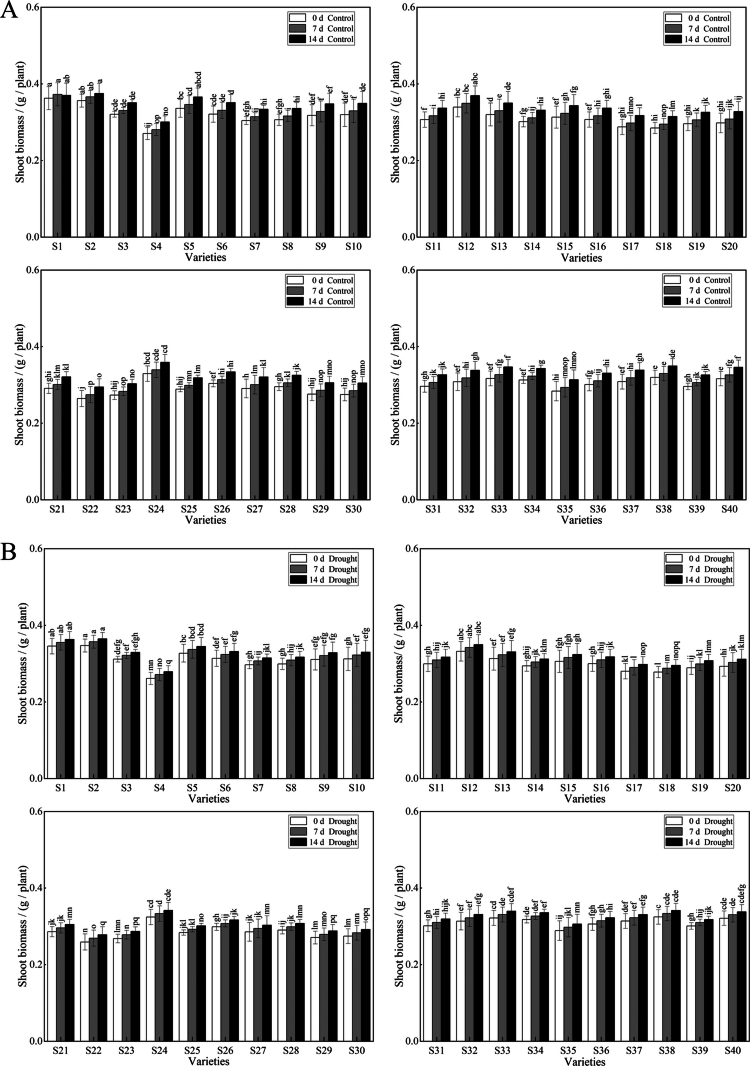
Effect of drought stress on the shoot biomass of different varieties of forage oats. Note: **(A)** represents the shoot biomass of the control. **(B)** represents the shoot biomass of the plants in the natural drought treatment groups. White color represents 0 d of drought stress. Gray color represents 7 d of drought stress. Black color represents 14 d of drought stress. Bars with different lowercase letters are significantly different at *P* < 0.05, and values represent the mean dry weight per plant ± standard error (Duncan's multiple range test).

**Figure 3.**
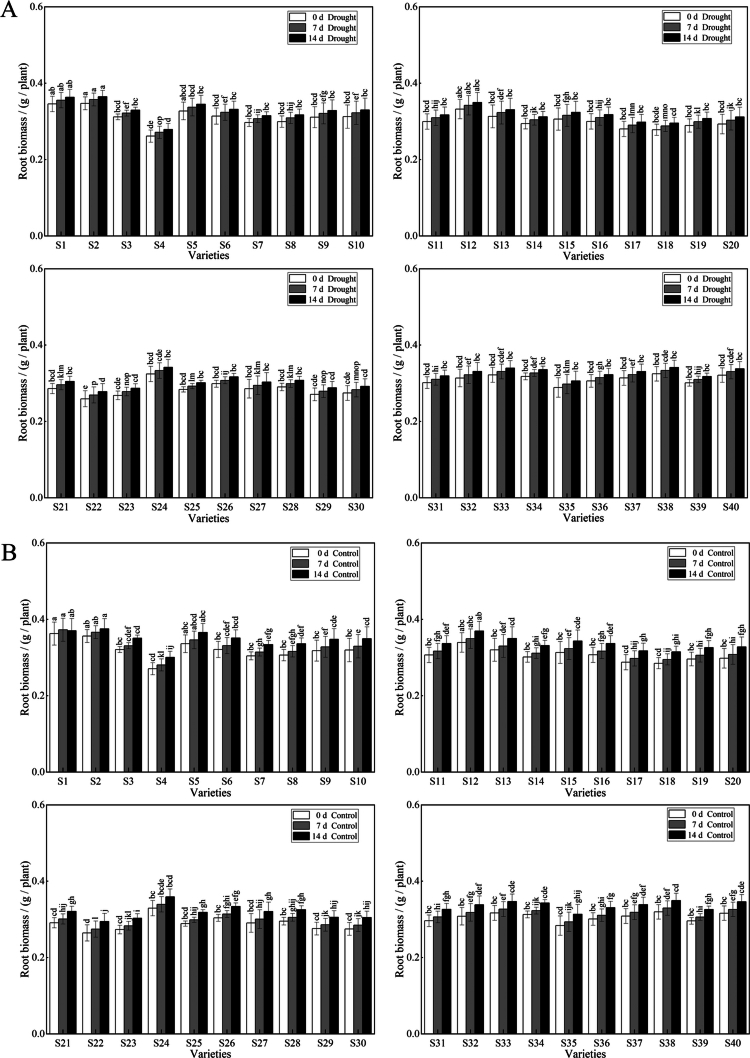
Effect of drought stress on the root biomass of different varieties of forage oats. Note: **(A)** represents the root biomass of the control. **(B)** represents the root biomass of the natural drought treatment groups. White color represents 0 d of drought stress. Gray color represents 7 d of drought stress. Black color represents 14 d of drought stress. Bars with different lowercase letters are significantly different at *P* < 0.05, and values represent the mean dry weight per plant ± standard error (Duncan's multiple range test).

Multiple comparisons and analysis of variance of the changes in forage oat biomass under different drought stress durations revealed that the forage oat shoot biomass (SB), although increasing as the duration of drought stress increased, was lower than that of the control (Figures 2 and 3). Following 14 d drought stress, S33 had the highest shoot biomass, while S4, S8, S10, S18, S20, S21, S23, S24, and S30 were significantly different from the control, with decreases of 29.70%, 32.28%, 29.04%, 29.45%, 31.47%, 31.33%, 34.19%, 33.48%, and 29.00%, respectively. The trend in the root biomass (RB) of the forage oats was similar to that in the shoot biomass (SB), and the RB of the drought-stressed plants was lower than that of the control plants. Compared with the control, S4 presented the greatest decrease of 7.12%, and the S1 variety presented the smallest decrease of 1.89%, indicating strong drought resistance.

When a plant experiences drought stress, its roots cannot obtain the water required for normal growth. Table 4 reported that the root-to-shoot ratio (RSR) of the oat cultivar tended to decrease with increasing drought stress. The RSR values of all the forage oats under drought stress were higher than those of the control. This indicated that when forage oats encountered drought stress, they automatically redistributed biomass by increasing the RSR to increase water uptake and optimize growth.

**Table 4.** Effect of drought stress on RSR of different cultivar of oat.

Cultivar	Control			Drought		
	0 d	7 d	14 d	0 d	7 d	14 d
S1	0.24 ± 0.07^ab^	0.29 ± 0.11^ab^	0.40 ± 0.11^ab^	0.24 ± 0.09^ab^	0.29 ± 0.14^ab^	0.18 ± 0.12^ab^
S2	0.26 ± 0.12^a^	0.30 ± 0.13^a^	0.41 ± 0.11^a^	0.26 ± 0.14^a^	0.3 ± 0.21^a^	0.19 ± 0.17^a^
S3	0.20 ± 0.04^abc^	0.26 ± 0.10^bcdefgh^	0.38 ± 0.13^abcde^	0.2 ± 0.05^bcdefgh^	0.26 ± 0.09^cdefg^	0.16 ± 0.07^abcde^
S4	0.22 ± 0.07^abc^	0.28 ± 0.12^abcd^	0.39 ± 0.13^abcde^	0.22 ± 0.08^bcd^	0.27 ± 0.15^bcd^	0.17 ± 0.13^abc^
S5	0.22 ± 0.08^abc^	0.27 ± 0.12^abcde^	0.38 ± 0.13^abcd^	0.22 ± 0.09^bcde^	0.26 ± 0.16^bcde^	0.16 ± 0.13^abcd^
S6	0.21 ± 0.12^abc^	0.27 ± 0.14^bcdefg^	0.38 ± 0.13^abcde^	0.21 ± 0.14^bcdefg^	0.26 ± 0.25^cdef^	0.16 ± 0.2^abcde^
S7	0.18 ± 0.04^abc^	0.25 ± 0.10^defghijk^	0.36 ± 0.13^abcde^	0.18 ± 0.05^defghijk^	0.24 ± 0.09^defghi^	0.14 ± 0.07^abcde^
S8	0.18 ± 0.07^abc^	0.25 ± 0.11^defghijk^	0.36 ± 0.09^abcde^	0.18 ± 0.08^defghijk^	0.24 ± 0.14^defghi^	0.14 ± 0.12^abcde^
S9	0.21 ± 0.17^abc^	0.27 ± 0.17^bcdefg^	0.38 ± 0.09^abcde^	0.21 ± 0.21^bcdefg^	0.25 ± 0.39^cdefghi^	0.16 ± 0.28^abcde^
S10	0.20 ± 0.12^abc^	0.26 ± 0.15^bcdefgh^	0.37 ± 0.09^abcde^	0.2 ± 0.15^bcdefgh^	0.24 ± 0.26^cdefghi^	0.15 ± 0.22^abcde^
S11	0.18 ± 0.10^abc^	0.25 ± 0.13^defghijk^	0.37 ± 0.09^abcde^	0.18 ± 0.12^defghijk^	0.23 ± 0.2^efghi^	0.15 ± 0.17^abcde^
S12	0.23 ± 0.15^abc^	0.28 ± 0.15^abc^	0.39 ± 0.09^abc^	0.23 ± 0.18^abc^	0.26 ± 0.29^bcde^	0.17 ± 0.23^abc^
S13	0.21 ± 0.16^abc^	0.27 ± 0.16^bcdef^	0.38 ± 0.09^abcde^	0.21 ± 0.2^bcdef^	0.25 ± 0.33^cdefghi^	0.16 ± 0.27^abcde^
S14	0.18 ± 0.05^abc^	0.25 ± 0.11^defghijk^	0.36 ± 0.09^abcde^	0.18 ± 0.06^defghijk^	0.23 ± 0.11^fghi^	0.14 ± 0.09^abcde^
S15	0.19 ± 0.11^abc^	0.26 ± 0.14^cdefghij^	0.37 ± 0.09^abcde^	0.19 ± 0.13^defghij^	0.24 ± 0.23^efghi^	0.15 ± 0.19^abcde^
S16	0.18 ± 0.09^abc^	0.25 ± 0.12^defghijk^	0.37 ± 0.11^abcde^	0.18 ± 0.1^defghijk^	0.23 ± 0.18^efghi^	0.15 ± 0.15^abcde^
S17	0.16 ± 0.07^bc^	0.24 ± 0.12^ghijk^	0.35 ± 0.11^bcde^	0.16 ± 0.08^ghijk^	0.22 ± 0.15^i^	0.13 ± 0.13^bcde^
S18	0.16 ± 0.05^bc^	0.23 ± 0.10^hijk^	0.35 ± 0.11^bcde^	0.16 ± 0.06^hijk^	0.22 ± 0.14^fghi^	0.13 ± 0.09^bcde^
S19	0.17 ± 0.07^abc^	0.24 ± 0.12^defghijk^	0.35 ± 0.11^cde^	0.17 ± 0.09^fghijk^	0.24 ± 0.15^cdefghi^	0.13 ± 0.12^cde^
S20	0.18 ± 0.09^abc^	0.24 ± 0.13^defghijk^	0.35 ± 0.11^bcde^	0.18 ± 0.11^defghijk^	0.25 ± 0.20^cdefghi^	0.13 ± 0.16^bcde^
S21	0.17 ± 0.05^bc^	0.24 ± 0.11^fghijk^	0.34 ± 0.11^cde^	0.17 ± 0.06^fghijk^	0.24 ± 0.11^defghi^	0.12 ± 0.09^cde^
S22	0.14 ± 0.06^c^	0.22 ± 0.11^k^	0.33 ± 0.11^e^	0.14 ± 0.07^k^	0.22 ± 0.13^ghi^	0.11 ± 0.11^e^
S23	0.15 ± 0.03^c^	0.23 ± 0.10^jk^	0.33 ± 0.11^e^	0.15 ± 0.04^jk^	0.23 ± 0.08^fghi^	0.11 ± 0.06^e^
S24	0.21 ± 0.08^abc^	0.27 ± 0.12^abcde^	0.37 ± 0.08^abcde^	0.21 ± 0.09^bcdef^	0.27 ± 0.16^abc^	0.15 ± 0.12^abcde^
S25	0.16 ± 0.02^bc^	0.24 ± 0.09^fghijk^	0.34 ± 0.03^cde^	0.16 ± 0.03^ghijk^	0.24 ± 0.05^defghi^	0.12 ± 0.04^cde^
S26	0.18 ± 0.04^abc^	0.25 ± 0.10^defghijk^	0.35 ± 0.05^bcde^	0.18 ± 0.05^defghijk^	0.25 ± 0.09^cdefg^	0.13 ± 0.07^bcde^
S27	0.17 ± 0.08^abc^	0.24 ± 0.13^defghijk^	0.34 ± 0.10^cde^	0.17 ± 0.1^fghijk^	0.24 ± 0.19^cdefghi^	0.12 ± 0.15^cde^
S28	0.17 ± 0.04^abc^	0.24 ± 0.10^defghijk^	0.35 ± 0.05^cde^	0.17 ± 0.05^fghijk^	0.25 ± 0.09^cdefghi^	0.13 ± 0.07^cde^
S29	0.15 ± 0.05^bc^	0.23 ± 0.11^ijk^	0.34 ± 0.07^de^	0.15 ± 0.06^ijk^	0.23 ± 0.12^fghi^	0.12 ± 0.11^de^
S30	0.15 ± 0.06^bc^	0.23 ± 0.11^ijk^	0.35 ± 0.07^cde^	0.15 ± 0.07^ijk^	0.23 ± 0.13^fghi^	0.13 ± 0.11^de^
S31	0.17 ± 0.06^abc^	0.25 ± 0.11^defghijk^	0.36 ± 0.07^abcde^	0.17 ± 0.07^efghijk^	0.22 ± 0.27^ghi^	0.14 ± 0.11^abcde^
S32	0.19 ± 0.08^abc^	0.26 ± 0.12^cdefghij^	0.37 ± 0.10^abcde^	0.19 ± 0.1^defghij^	0.22 ± 0.18^hi^	0.15 ± 0.15^abcde^
S33	0.20 ± 0.09^abc^	0.27 ± 0.13^bcdefg^	0.38 ± 0.11^abcde^	0.2 ± 0.11^bcdefgh^	0.23 ± 0.2^fghi^	0.16 ± 0.16^abcde^
S34	0.19 ± 0.04^abc^	0.26 ± 0.10^bcdefghij^	0.37 ± 0.05^abcde^	0.19 ± 0.05^cdefghi^	0.24 ± 0.09^defghi^	0.15 ± 0.07^abcde^
S35	0.16 ± 0.08^bc^	0.24 ± 0.12^fghijk^	0.35 ± 0.10^bcde^	0.16 ± 0.1^hijk^	0.22 ± 0.18^hi^	0.13 ± 0.15^bcde^
S36	0.18 ± 0.07^abc^	0.25 ± 0.12^defghijk^	0.36 ± 0.08^abcde^	0.18 ± 0.08^defghijk^	0.23 ± 0.15^fghi^	0.14 ± 0.12^abcde^
S37	0.19 ± 0.10^abc^	0.26 ± 0.13^cdefghij^	0.37 ± 0.11^abcde^	0.19 ± 0.12^defghij^	0.24 ± 0.21^efghi^	0.15 ± 0.17^abcde^
S38	0.20 ± 0.07^abc^	0.26 ± 0.11^bcdefgh^	0.37 ± 0.08^abcde^	0.2 ± 0.08^cdefgh^	0.24 ± 0.14^cdefghi^	0.15 ± 0.12^abcde^
S39	0.17 ± 0.04^abc^	0.24 ± 0.10^defghijk^	0.36 ± 0.04^abcde^	0.17 ± 0.04^fghijk^	0.23 ± 0.08^fghi^	0.14 ± 0.07^abcde^
S40	0.20 ± 0.09^abc^	0.26 ± 0.13^bcdefg^	0.37 ± 0.10^abcde^	0.2 ± 0.11^bcdefgh^	0.25 ± 0.19^cdefghi^	0.15 ± 0.15^abcde^

Note: Values with different superscript letters (a, b, c) within a row indicate significant differences (*P* < 0.05) across timepoints (Duncan's multiple range test).

The relative water content (RWC) is the basis for maintaining the normal physiological role of the plant body. The level of RWC can reflect the strength of plant leaf water retention capacity. Table 5 reported that the leaf moisture content of forage oat in the control group was greater than 91%, and the drought treatment group presented a decreasing trend in leaf moisture content, of which S4, S14, S17, S18, S19, S20, S21, S22, S23, S29, and S30 presented the greatest decreases. Under 14 d of drought stress, S28 had the highest RWC, followed by S2, S5, and S33, indicating that the LWC of these cultivars was strongest under the drought-stress treatments.

**Table 5.** Effect of drought stress on relative water content of different cultivar of oat.

Cultivar	Control			Drought		
	0 d	7 d	14 d	0 d	7 d	14 d
S1	0.92 ± 0.13^abcd^	0.91 ± 0.01^ab^	0.90 ± 0.01^a^	0.94 ± 0.02^abcd^	0.86 ± 0.24^abcd^	0.69 ± 0.17^abcd^
S2	0.93 ± 0.02^a^	0.91 ± 0.02^a^	0.90 ± 0.02^ab^	0.94 ± 0.02^a^	0.87 ± 0.2^a^	0.70 ± 0.14^ab^
S3	0.93 ± 0.01^abcdef^	0.91 ± 0.02^abc^	0.90 ± 0.02^abcd^	0.93 ± 0.01^abcde^	0.86 ± 0.14^abc^	0.68 ± 0.12^abc^
S4	0.92 ± 0.22^i^	0.89 ± 0.03^bcd^	0.88 ± 0.02^cde^	0.91 ± 0.02^h^	0.83 ± 0.46^e^	0.64 ± 0.42^f^
S5	0.93 ± 0.02^abc^	0.90 ± 0.03^abcd^	0.90 ± 0.02^abcd^	0.94 ± 0.02^abcd^	0.87 ± 0.19^abc^	0.70 ± 0.16^ab^
S6	0.94 ± 0.03^abcde^	0.89 ± 0.03^abcd^	0.88 ± 0.02^abcde^	0.93 ± 0.03^abcd^	0.85 ± 0.25^abcde^	0.68 ± 0.19^abcdef^
S7	0.92 ± 0.01^bcdefghi^	0.90 ± 0.03^abcd^	0.88 ± 0.03^abcde^	0.93 ± 0.01^bcdefgh^	0.85 ± 0.15^abcde^	0.68 ± 0.13^bcdef^
S8	0.92 ± 0.02^bcdefghi^	0.89 ± 0.02^abcd^	0.88 ± 0.01^bcde^	0.93 ± 0.02^bcdefg^	0.85 ± 0.2^abcde^	0.68 ± 0.17^abcdef^
S9	0.92 ± 0.04^abcdefg^	0.90 ± 0.04^abcd^	0.89 ± 0.03^abcde^	0.93 ± 0.14^abcde^	0.86 ± 0.3^abc^	0.69 ± 0.22^abc^
S10	0.92 ± 0.03^abcdefg^	0.90 ± 0.04^abcd^	0.89 ± 0.02^abcde^	0.93 ± 0.03^bcdef^	0.85 ± 0.29^abcde^	0.68 ± 0.23^abcdef^
S11	0.92 ± 0.02^bcdefghi^	0.90 ± 0.02^abcd^	0.89 ± 0.02^abcde^	0.93 ± 0.22^bcdefg^	0.86 ± 0.21^abcde^	0.68 ± 0.16^abcdef^
S12	0.93 ± 0.03^ab^	0.91 ± 0.02	0.90 ± 0.02^abc^	0.94 ± 0.03^ab^	0.86 ± 0.32^abc^	0.69 ± 0.25^abc^
S13	0.93 ± 0.34^abcde^	0.91 ± 0.03^abcd^	0.90 ± 0.02^abc^	0.93 ± 0.04^abcd^	0.86 ± 0.18^abcd^	0.69 ± 0.11^abcd^
S14	0.93 ± 0.02^cdefghu^	0.90 ± 0.03^abcd^	0.89 ± 0.01^abcde^	0.93 ± 0.02^cdefgh^	0.85 ± 0.16^bcde^	0.67 ± 0.13^bcdef^
S15	0.93 ± 0.03^bcdefghi^	0.90 ± 0.03^abcd^	0.89 ± 0.02^abcde^	0.93 ± 0.03^bcdefg^	0.86 ± 0.17^abc^	0.69 ± 0.16^abc^
S16	0.93 ± 0.52^bcdefghi^	0.91 ± 0.01^abcd^	0.90 ± 0.01^abcd^	0.93 ± 0.02^bcdefg^	0.86 ± 0.2^abcde^	0.68 ± 0.16^abcde^
S17	0.91 ± 0.02^efghi^	0.90 ± 0.03^abcd^	0.89 ± 0.02^abcde^	0.92 ± 0.02^efgh^	0.85 ± 0.37^bcde^	0.67 ± 0.31^bcdef^
S18	0.92 ± 0.52^ghi^	0.90 ± 0.03^abcd^	0.89 ± 0.02^bcde^	0.92 ± 0.12^fgh^	0.85 ± 0.19^cde^	0.67 ± 0.15^bcdef^
S19	0.92 ± 0.02^cdefghi^	0.90 ± 0.03^abcd^	0.88 ± 0.01^bcde^	0.93 ± 0.02^cdefgh^	0.85 ± 0.22^bcde^	0.67 ± 0.18^bcdef^
S20	0.93 ± 0.43^cdefghi^	0.90 ± 0.02^abcd^	0.89 ± 0.02^abcde^	0.93 ± 0.03^cdefgh^	0.85 ± 0.32^bcde^	0.67 ± 0.26^bcdef^
S21	0.92 ± 0.02^cdefghi^	0.9 ± 0.03^cd^	0.88 ± 0.01^cde^	0.93 ± 0.02^cdefgh^	0.84 ± 0.19^cde^	0.67 ± 0.16^cdef^
S22	0.92 ± 0.02^i^	0.91 ± 0.02^cd^	0.88 ± 0.02^de^	0.92 ± 0.52^h^	0.83 ± 0.37^e^	0.65 ± 0.32^ef^
S23	0.92 ± 0.01^hi^	0.9 ± 0.03^d^	0.88 ± 0.02^e^	0.92 ± 0.01^gh^	0.84 ± 0.22^cde^	0.66 ± 0.2^def^
S24	0.93 ± 0.02^abcd^	0.89 ± 0.02^cd^	0.89 ± 0.02^abcde^	0.94 ± 0.02^abc^	0.85 ± 0.24^abcde^	0.68 ± 0.17^abcdef^
S25	0.93 ± 0.01^defghi^	0.90 ± 0.02^abcd^	0.89 ± 0.02^abcde^	0.92 ± 0.01^defgh^	0.85 ± 0.23^abcde^	0.68 ± 0.2^bcdef^
S26	0.92 ± 0.01^bcdefgh^	0.90 ± 0.03^abcd^	0.89 ± 0.02^abcde^	0.93 ± 0.01^bcdefg^	0.86 ± 0.22^abcde^	0.68 ± 0.19^abcd^
S27	0.92 ± 0.03^cdefghi^	0.90 ± 0.02^bcd^	0.88 ± 0.02^abcde^	0.93 ± 0.03^cdefgh^	0.86 ± 0.15^abcd^	0.69 ± 0.12^abcd^
S28	0.93 ± 0.01^cdefghi^	0.91 ± 0.02^abcd^	0.89 ± 0.01^abcde^	0.93 ± 0.01^cdefgh^	0.85 ± 0.19^abcde^	0.71 ± 0.16^a^
S29	0.91 ± 0.02^hi^	0.90 ± 0.01^abcd^	0.89 ± 0.01^abcde^	0.92 ± 0.02^gh^	0.85 ± 0.26^bcde^	0.67 ± 0.22^bcdef^
S30	0.91 ± 0.42^hi^	0.89 ± 0.02^cd^	0.89 ± 0.03^abcde^	0.92 ± 0.02^gh^	0.85 ± 0.16^bcde^	0.67 ± 0.12^bcdef^
S31	0.92 ± 0.02^cdefghi^	0.90 ± 0.02^cd^	0.89 ± 0.02^abcde^	0.93 ± 0.02^cdefgh^	0.86 ± 0.17^abc^	0.69 ± 0.14^abc^
S32	0.92 ± 0.62^bcdefgh^	0.90 ± 0.01^cd^	0.90 ± 0.01^abcd^	0.93 ± 0.12^bcdefg^	0.86 ± 0.18^abc^	0.69 ± 0.15^abc^
S33	0.93 ± 0.02^abcdef^	0.91 ± 0.01^abc^	0.90 ± 0.02^abc^	0.93 ± 0.02^abcde^	0.87 ± 0.19^abc^	0.70 ± 0.15^abc^
S34	0.92 ± 0.01^bcdefg^	0.91 ± 0.01^abcd^	0.90 ± 0.01^abcd^	0.93 ± 0.01^bcdef^	0.86 ± 0.21^abcd^	0.69 ± 0.18^abcd^
S35	0.92 ± 0.33^fghi^	0.90 ± 0.02^cd^	0.89 ± 0.02^abcde^	0.92 ± 0.03^efgh^	0.86 ± 0.19^abcd^	0.69 ± 0.15^abcd^
S36	0.93 ± 0.32^bcdefghi^	0.92 ± 0.03^abcd^	0.89 ± 0.03^abcde^	0.93 ± 0.32^bcdefgh^	0.86 ± 0.17^abcde^	0.68 ± 0.14^bcdef^
S37	0.93 ± 0.02^bcdefgh^	0.90 ± 0.02^cd^	0.89 ± 0.01^abcde^	0.93 ± 0.02^bcdefg^	0.86 ± 0.19^abcd^	0.69 ± 0.14^abcd^
S38	0.93 ± 0.12^bcdefg^	0.92 ± 0.02^abcd^	0.89 ± 0.01^abcde^	0.93 ± 0.02^bcdef^	0.85 ± 0.21^abcd^	0.69 ± 0.16^abcd^
S39	0.92 ± 0.01^cdefghi^	0.90 ± 0.01^cd^	0.89 ± 0.02^abcde^	0.93 ± 0.01^cdefgh^	0.86 ± 0.18^abc^	0.69 ± 0.15^abc^
S40	0.94 ± 0.02^ab^	0.91 ± 0.02^bcd^	0.90 ± 0.02^abcde^	0.93 ± 0.02^abcde^	0.85 ± 0.14^abc^	0.69 ± 0.11^abc^

Note: Values with different superscript letters (a, b, c) within a row indicate significant differences (*P* < 0.05) across timepoints (Duncan's multiple range test).

### 3.3 Correlation analysis of phenotypic indicators of drought resistance in forage oat

**Figure 4.**
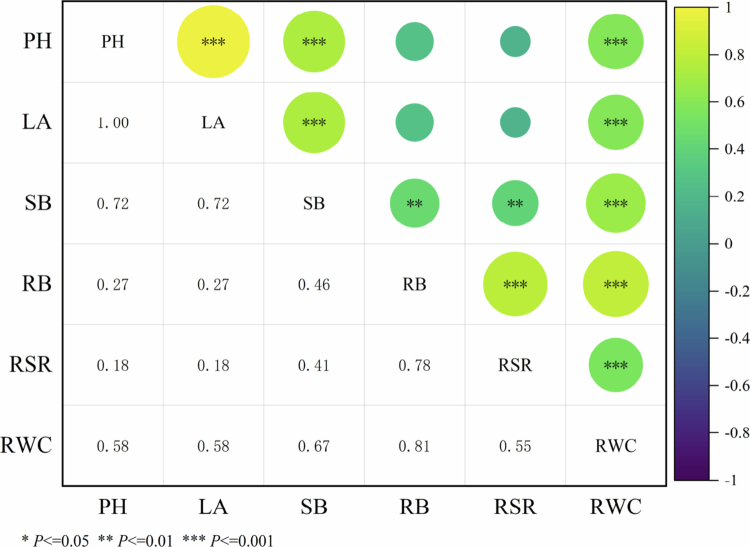
Correlations between growth phenotype indicators. Note: PH, plant height; LA, leaf area; SB, shoot biomass; RB, root biomass; RWC, relative water content; RSR, root-to-shoot ratio.

A correlation analysis of the six measurement indices was performed (Figure 4). The results revealed that PH was significantly positively correlated with LA, SB and RWC. LA was significantly positively correlated with RWC, SB was significantly positively correlated with RWC, RB was significantly positively correlated with RSR and RWC, and RSR was significantly positively correlated with RWC.

### 3.4 Comprehensive evaluation of drought resistance in different cultivar of forage oat

Because the phenotypic indicators were closely related and contained overlapping information, the main influencing factors were extracted through principal component analysis to reduce the dimensionality of each indicator. The results revealed that the first principal component contributed 63.11% of the variance and that the second principal component contributed 25.24% (Table 6), so the cumulative variance contribution rate of the first two principal components was 88.35%. Therefore, these components effectively reflected most of the information of the six measured indicators.

**Table 6.** Characteristic value and cumulative contribution rate of individual factors.

Index	PCⅠ	PCⅡ
Plant height	0.216	−0.354
Single leaf area	0.216	−0.354
Shoot biomass	0.227	−0.120
Root biomass	0.193	0.410
Root-to-shoot ratio	0.158	0.462
Relative water content	0.238	0.120
Eigenvalue	3.786	1.514
Variance contribution rate/%	63.107	25.235
Cumulative contribution rate/%	63.107	88.342

Therefore, the six measured indices were transformed into two independent composite indices. These values were used in the next step of calculating the comprehensive evaluation value for forage oat.

The D-value is the sum of the values of the affiliation function of each comprehensive index, reflecting the drought resistance strength of each test material. The larger the D-value is, the higher the drought resistance; the smaller the D-value is, the lower the drought resistance. The D-value of each forage oat variety was calculated, and the drought resistance of the 40 test materials was ranked according to the magnitude of the D-value. Table 7 reported that the maximum value of the weighted affiliation function was 0.5978, corresponding to variety AN207 and indicating that this variety was the most drought resistant. The smallest weighted affiliation function value of 0.1009 corresponded to variety IN01, indicating that this variety was the least drought resistant.

**Figure 5.**
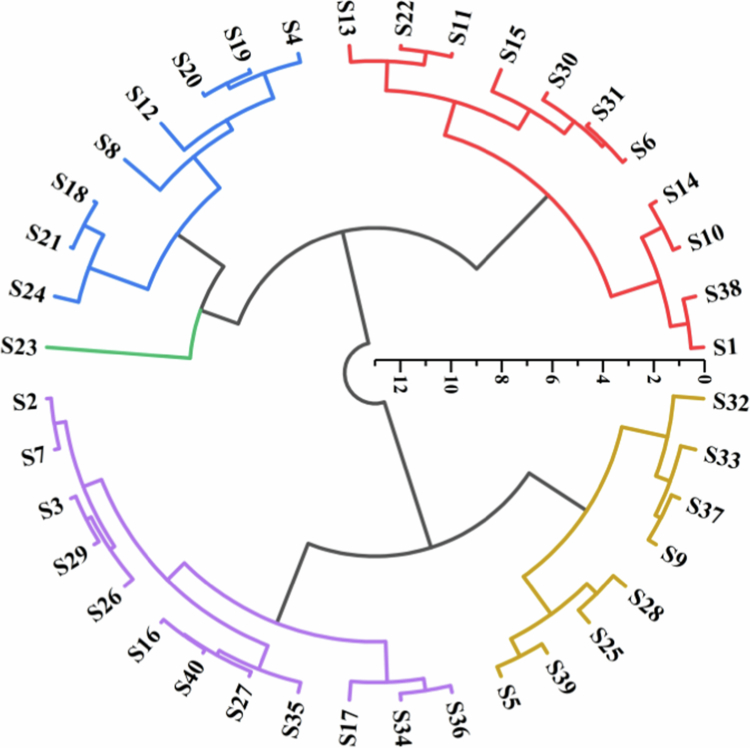
Cluster analysis plot of 40 forage oat varieties. Note: Red, for highly drought resistant plants. Purple, for medium resistant plants. Blue, for low resistance plants. Yellow, for low susceptible plants. Green, for highly susceptible plants.

The 40 forage oat cultivars were analyzed by clustering (Figure 5). The results revealed five classifications of drought resistance, with Class I having high resistance, Class II having moderate resistance, Class III having low resistance, Class IV having low susceptibility, and Class V having high susceptibility. A representative oat variety was selected from each of the five drought-resistant types, and its photosynthetic and physiological indices were compared.

### 3.5 Analysis of changes in biochemical parameters of forage oat under drought stress

**Figure 6.**
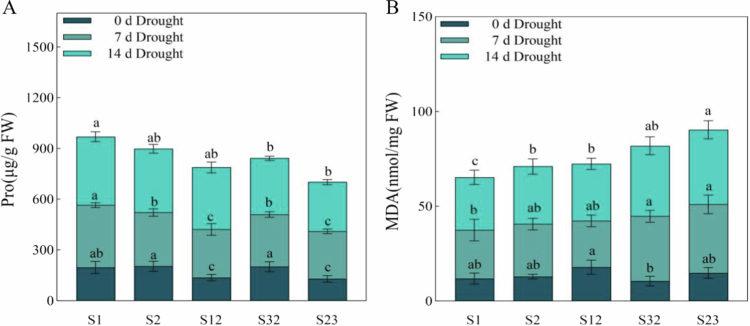
Changes in Pro and MDA contents in oat leaves under drought stress. Note: (**A**) Variations in and comparisons of the Pro contents of five types of forage oats. (**B**) Variations in and comparisons of the MDA contents of five types of forage oats. Dark blue represents 0 d of drought stress. Grayish blue represents 7 d of drought stress. Sky blue represents 14 d of drought stress. Bars with different lowercase letters are significantly different at *P* < 0.05, and values represent the mean dry weight per plant ± standard error (Duncan's multiple range test).

When plants are subjected to drought stress, they can adapt by regulating their nonstructural carbohydrate content. It was found that proline (Pro) content increased with increasing drought stress. Under the 7 d drought stress treatment, S1 presented the highest Pro content, whereas S12 and S23 presented the lowest Pro content (Figure 6A). Under 14 d of drought stress, the Pro contents of S1, S2, and S3 increased significantly, but the contents of S32 and S23 did not change much compared with those under 7 d of drought stress. The Pro content of S1 remained the highest. Compared with that in the control, the malondialdehyde (MDA) content in the leaves of the forage oat plants changed the most in S32 and S23 under the 7 d of drought stress treatment. The MDA content was significantly increased in all forage oat leaves under the 14 d drought stress treatment (Figure 6B), indicating increased reactive oxygen species (ROS) damage in forage oat leaves during severe drought.

**Figure 7.**
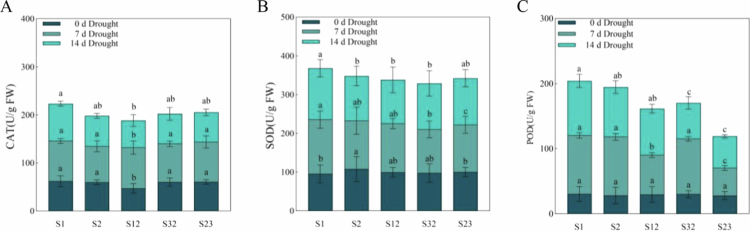
Antioxidant enzyme activity content of oat under drought stress. Note: (**A**) Variations in and comparisons of the CAT contents of five types of forage oats. (**B**) Variations in and comparisons of the SOD contents of the five types of forage oats. (**C**) Variation in and comparison of POD contents among the five types of forage oats. Dark blue represents 0 d of drought stress. Grayish blue represents 7 d of drought stress. Sky blue represents 14 d of drought stress. Bars with different lowercase letters are significantly different at *P* < 0.05, and values represent the mean dry weight per plant ± standard error (Duncan's multiple range test).

Antioxidant enzymes can scavenge free oxygen species in plants and mitigate damage to membrane systems, with the strength of their activity corresponding to the adaptability of the plant to adverse conditions. The contents of the three antioxidant enzymes did not differ significantly under the 0 d drought stress treatment (Figure 7). The activity of all three antioxidant enzymes increased under the 7 d drought stress treatment, with little difference in the changes in catalase (CAT) activity and superoxide dismutase (SOD) activity and significant differences in the increase in peroxidase (POD) activity among the five oat cultivars. The lowest change in POD activity was observed in S23. Under the 14 d drought stress treatment, the CAT and POD activities of the five different drought-resistant oat cultivars significantly decreased, and the change in SOD activity also tended to decrease, but this change was not pronounced. The POD activity of the five different oat cultivars varied significantly, with S23 having the lowest POD content.

### 3.6 Analysis of changes in photosynthetic characteristics of forage oat under drought stress

**Figure 8.**
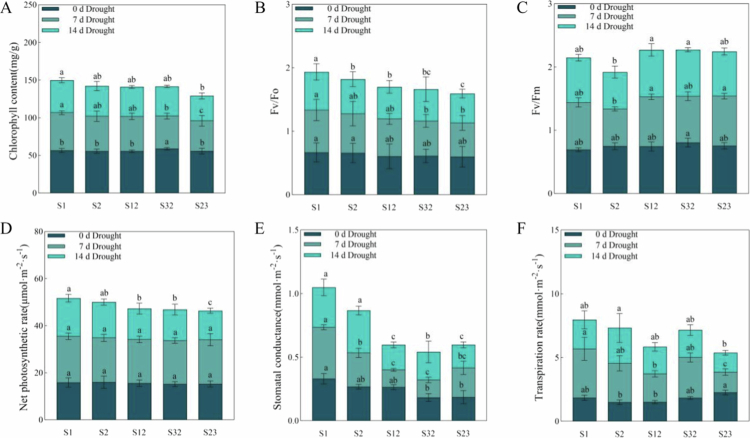
Analysis of changes in the photosynthetic characteristics of forage oats under drought stress. Note: (**A**) Variation and comparison of the chlorophyll content for five types of forage oat. (**B**) Variations in and comparisons of the Fv/Fo contents of five types of forage oats. (**C**) Variations in and comparisons of the Fv/Fm contents of five types of forage oats. (**D**) Variations in and comparisons of the Pn contents of five types of forage oats. (**E**) Variations in and comparisons of the Gs contents of five types of forage oats. (**F**) Variations in and comparisons of the Tr contents of five types of forage oats. Dark blue represents 0 d of drought stress. Grayish blue represents 7 d of drought stress. Sky blue represents 14 d of drought stress. Bars with different lowercase letters are significantly different at *P* < 0.05, and values represent the mean dry weight per plant ± standard error (Duncan's multiple range test).

Photosynthesis is the main process by which plants obtain a source of energy during growth. Drought stress alters photosynthesis in plants by affecting the chlorophyll content and altering transpiration and stomatal conductance. Different drought durations significantly affected the chlorophyll (Chl) content and the PS II quantum efficiency (Fv/Fo), maximum photochemical quantum yield (Fv/Fm), transpiration rate (Tr), net photosynthetic rate (Pn), and stomatal conductance (Gs) values in oat leaves (*P* < 0.05). Under the 14 d drought treatment, the oat leaves yellowed, the leaf stems wilted, and the Chl content of the oat leaves decreased (Figure 8A). S1 had the highest Chl content, and S23 had the lowest Chl content under the 14 d drought treatment.

In the chlorophyll fluorescence parameter assay, Fv/Fo represents photosystem II (PS II) activity, and Fv/Fm represents the maximum efficiency of photosystem II photochemistry. Compared with those under 0 d drought stress, the plant Fv/Fo values under 14 d drought stress decreased (Figure 8B), and the PS II activity was greatest in S1 and lowest in S23. Similarly, under 14 d drought stress, the Fv/Fm values decreased (Figure 8C). However, S12 had the highest photosystem II photochemistry efficiency and S2 had the lowest.

Under drought stress, the Tr (Figure 8D), Pn (Figure 8E), and Gs (Figure 8F) of the plants were significantly reduced (*P* < 0.05), with all increasing and then decreasing as the drought stress duration increased.

### 3.7 Principal component analysis of physiological indices in fed oat

**Figure 9.**
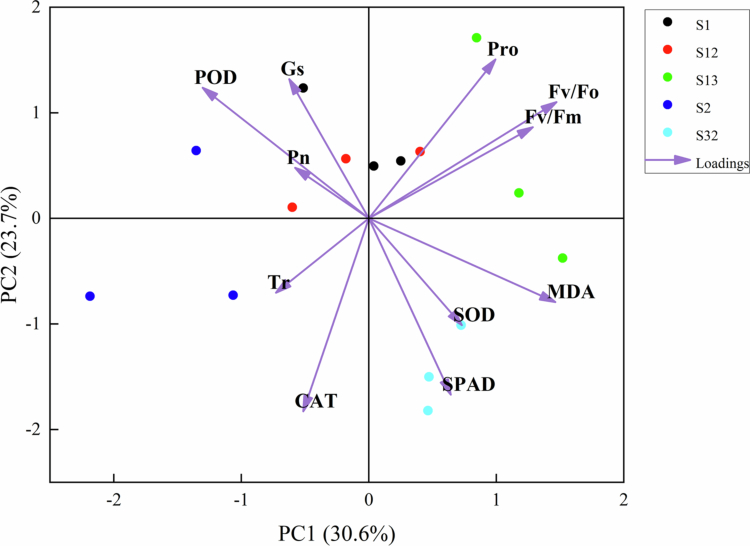
PCA of physiological indices of oat under drought stress. Note: Black dots represent S1, red dots represent S2, green dots represent S13, dark blue dots represent S2, and bright blue dots represent S32.

As shown in Figure 9, the PCA of the drought biochemical parameters measured for oat revealed that the cumulative contribution of the first five principal components was 86.49%, and the 11 drought biochemical parameters could be replaced by these five principal components. Among the five principal components, Fv/Fo, Pro, Tr, Pn and Gs had the highest factor loadings. Therefore, these five indicators could be used as the main drought resistance assessment indicators for oat during drought stress.

## 4 Discussion

Climate change-induced extreme weather events fundamentally alter abiotic conditions for plant survival, with water scarcity emerging as a critical limiting factor for global plant productivity and food security.^31^^,^^32^ While plant responses to drought involve complex interactions across growth, physiological, and biochemical levels,^33^ this study establishes that integrated phenotypic screening provides a robust approach for identifying drought-tolerant forage oat cultivars—a vital strategy for sustainable agriculture in water-limited environments.

### 4.1 Phenotypic indicators as primary screening tools

Consistent with observations in soybean^34^ and maize,^35^ drought stress universally inhibited growth, including reduced plant height (PH), leaf area (LA), biomass and relative water content (RWC), across 40 forage oat cultivars. Crucially, however, our cluster analysis demonstrated that specific phenotypic traits serve as highly effective, rapid indicators for preliminary drought tolerance screening in oats. This contrasts with studies focused solely on complex physiological assays,^36^^,^^37^ providing a practical, high-throughput field selection method.

### 4.2 Photosynthetic decline and tolerance mechanisms

Photosynthesis is a determinant of plant growth and productivity,^38^ with crop yield directly determined by photosynthesis. Drought stress causes deficiencies in certain elements in plants, leading to a lower rate of chlorophyll synthesis and a decrease in chlorophyll content within the plants.^3^ In the present study, as the degree of drought stress increased, photosynthetic parameters such as the net photosynthetic rate, transpiration rate, and stomatal conductance decreased, which was consistent with studies on wheat,^39^ maize,^40^ and cucumber.^35^ However, the magnitude of the decline differed for different oat cultivars. The size of plant leaf stomata, to some extent, determines the rate of transpiration, which is the most critical variable affecting the photosynthetic capacity of plants under drought treatment.^41^ Therefore, stomata play a vital role in maintaining the water balance in plants under drought stress. Research has shown that under drought stress, plant stomata close, and stomatal conductance is affected,^42^ which aligns with the findings of this study. A reduction in Gs leads to a decrease in the availability of CO_2_ for plants, resulting in a decrease in the rate of photosynthesis. Consequently, under drought stress, the CO_2_ photosynthetic rate of 40 forage oat cultivars decreased. However, drought-tolerant plants present a greater photosynthetic rate than do drought-sensitive plants. The inhibition of plant height and root length growth and the reduction in biomass may be attributed to the decreased CO_2_ assimilation rate, leading to a reduction in photosynthetic products.

### 4.3 PSII integrity and biomass linkage

Chlorophyll fluorescence parameters can indirectly reflect the photosynthetic capacity of plants.^43^ Fv/Fm, a photochemical quenching parameter, determines the maximum quantum efficiency of dark reactions. In the present study, under drought stress, the Fv/Fm and Fv/Fo tended to decrease as drought stress increased compared with those of the control, and the decrease was exacerbated as the duration of drought stress increased. These results are consistent with those for maize^44^ and potato,^45^ indicating that drought stress reduces the photochemical conversion efficiency of PSII in plants, leading to a decline in electron transport capacity, reduced photosynthetic efficiency, and ultimately affecting plant biomass. The decline in Fv/Fm was more pronounced in highly drought-sensitive plants than in highly drought-resistant plants, indicating that drought stress resulted in damage to the PSII reaction center in the leaves of the forage oat, resulting in decreased photosynthesis, decreased photosynthetic product production and—ultimately—decreased plant biomass.

### 4.4 Oxidative stress markers and defense strategies

The MDA content is an important parameter reflecting the damage caused by drought stress in plants. Several studies have shown that the MDA content in plants is closely related to plant stress tolerance.^46^^,^^47^ When plants are subjected to drought stress, their cell membranes are damaged first. The oxidative decomposition of cell membranes occurs, leading to a gradual increase in the MDA content, which further leads to plant metabolic disorders, a large amount of cellular content exocytosis and degradation, and, ultimately, cell death.^14^ In this study, the MDA content in the plants increased as the duration of drought stress increased and was greater than that in the control. The difference in the MDA content among the five forage oat cultivars was significant (*P* < 0.05), and there was a significant correlation between the MDA content and several other indices. These findings indicate that the MDA content is important for evaluating drought resistance, which is consistent with the results of studies on wheat and winter wheat.^48^^,^^49^ Pro is a compatible solute that helps enhance plant resistance under abiotic stress conditions. Additionally, Pro plays a positive role in scavenging ROS and mitigating damage to cell membranes during drought stress.^50^ Under normal conditions, the Pro content in plants is low. Under drought stress, the Pro content in plants increases, with a greater increase indicating greater drought resistance.^51^ Under drought stress, the Pro content of the five oat materials was significantly elevated, and the differences among the five oat cultivars were significant, which is consistent with the results of Chen.^52^

Drought stress, while increasing cellular ROS levels, can also promote the establishment of plant defense systems to prevent or slow down damage caused by ROS in plants.^53^^,^^54^ The antioxidant system is one of the main defense systems of plants, and SOD, CAT, and POD are the most important antioxidant enzymes.^54^ SOD converts O₂⁻ into H₂O₂ through a dismutation reaction. Since H₂O₂ is an ROS product, under drought stress, relying solely on SOD activity is insufficient to protect plants from oxidative damage. It must be further scavenged by CAT and POD to increase plant drought resistance.^53^ Drought stress significantly increased the activity of protective enzymes in wheat seedlings and tended to increase with increasing duration of stress.^55^ Partially similar results were obtained in the present study. Drought-tolerant plants accumulate only large quantities of osmoregulatory substances at the beginning of drought stress, followed by a gradual increase in antioxidant enzyme accumulation only with the intensification of drought. In contrast, drought-sensitive plants begin to accumulate antioxidant enzymes at the beginning of mild drought, while most of their protective mechanisms stop or slow under severe drought conditions.^56^ In this study, the antioxidant enzyme activities of the plants slightly changed in the early stages of drought stress but tended to increase, which may be due to the accumulation of antioxidant enzymes. As the duration of drought stress increased, the antioxidant enzyme activity partially decreased, with CAT and SOD activities decreasing insignificantly and POD activity significantly decreasing.

### 4.5 Multidimensional drought resistance evaluation using PCA-based clustering

Plants respond differently to drought stress because of differences in plant species, the degree of drought stress, the environment in which they grow, and their growth stage.^57^^,^^58^ Therefore, a single evaluation method cannot completely identify and evaluate the drought resistance of plants, and it is necessary to scientifically and systematically assess multiple indicators. Researchers have increasingly used drought resistance coefficients, PCA, correlation function analysis, factor analysis, regression analysis, gray correlation analysis, cluster analysis, and other methods to comprehensively evaluate the drought resistance of different plant species. In this study, six growth indicators were divided into two principal component factors using principal component analysis, with a cumulative contribution of 88.342%. Forty forage oat materials were categorized into five drought-resistant categories, reflecting the differences in their drought resistance.

## 5 Conclusion

In this study, 40 forage oat cultivars were subjected to drought stress treatment, and the comprehensive drought resistance rating (D-value) was used to classify the forage oat seedlings into five drought resistance levels. Based on the D-value as the primary indicator, five representative cultivars with different drought resistance levels were selected. This study revealed that the tolerant genotype (S1) can withstand drought conditions through its efficient photosynthetic system and the activity of antioxidant enzymes. Compared with the sensitive genotype (S23), S1 forms a more robust protective system against damage caused by drought. PCA revealed Fv/Fo, Pro, Tr, Gs, and Pn as the main indicators of the response of forage oats to drought. Changes in the content of osmoregulatory substances and increased activity of antioxidant enzymes may be important factors in the response of forage oats to drought stress. To evaluate the drought resistance of forage oats through their photosynthetic and other biochemical parameters, it is necessary to utilize a variety of indicators and methods to conduct a comprehensive analysis. This evaluation method can be used as a reference for screening good drought-tolerant cultivars to clarify the physiological differences in the response of different drought-resistant oat cultivars to water stress and to provide a theoretical basis for screening drought-resistant cultivars and achieving high-yield oat cultivation.

**Table 7.** D-values and comprehensive ranking of 40 forage oat cultivar.

Cultivar	μ1	μ2	D-values	Drought resistance
S1	0.9622	0.9740	0.9655	1
S2	0.8486	0.9199	0.8689	2
S3	0.7773	0.5661	0.7173	6
S4	0.2153	0.6180	0.3298	37
S5	1.0000	0.4803	0.8523	3
S6	0.5279	0.7580	0.5933	17
S7	0.5658	0.3547	0.5058	25
S8	0.3297	0.7800	0.4577	29
S9	0.8175	0.4645	0.7171	7
S10	0.5415	0.6503	0.5724	19
S11	0.5039	0.6790	0.5536	20
S12	0.6618	1.0000	0.7579	5
S13	0.6271	0.7872	0.6726	13
S14	0.4568	0.4519	0.4554	30
S15	0.6595	0.6235	0.6493	14
S16	0.7162	0.3892	0.6232	16
S17	0.5184	0.2379	0.4387	31
S18	0.2203	0.6309	0.3370	36
S19	0.2872	0.5902	0.3733	33
S20	0.3133	0.5773	0.3883	32
S21	0.1893	0.6367	0.3164	38
S22	0.1544	0.1730	0.1597	39
S23	0.0000	0.5483	0.1559	40
S24	0.3766	0.9717	0.5458	23
S25	0.6489	0.0000	0.4644	27
S26	0.5971	0.3430	0.5249	24
S27	0.5700	0.1890	0.4617	28
S28	0.6580	0.1176	0.5044	26
S29	0.4653	0.0996	0.3614	34
S30	0.3544	0.3433	0.3513	35
S31	0.5862	0.5759	0.5833	18
S32	0.8169	0.4135	0.7022	9
S33	0.9752	0.4998	0.8401	4
S34	0.7422	0.5984	0.7013	10
S35	0.6707	0.2577	0.5533	21
S36	0.5971	0.4337	0.5506	22
S37	0.8230	0.3836	0.6981	11
S38	0.7129	0.6490	0.6947	12
S39	0.8253	0.2012	0.6479	15
S40	0.7885	0.5246	0.7135	8
